# Some environmental and biological determinants of coral richness, resilience and reef building in Galápagos (Ecuador)

**DOI:** 10.1038/s41598-019-46607-9

**Published:** 2019-07-16

**Authors:** Bernhard Riegl, Matthew Johnston, Peter W. Glynn, Inti Keith, Fernando Rivera, Mariana Vera-Zambrano, Stuart Banks, Joshua Feingold, Peter J. Glynn

**Affiliations:** 1 Halmos College of Natural Science and Oceanography, Nova Southeastern University, 8000 N Ocean Drive, Dania Beach, Florida 33004 USA; 2 Rosenstiel School of Marine and Atmospheric Science, University of Miami, 4600 Rickenbacker Causeway, Miami, Florida 33149-1098 USA; 3 Fundación Charles Darwin, Charles Darwin Research Station, Puerto Ayora, Santa Cruz Island, 200350 Galápagos, Ecuador; 4 Instituto Nazca de Investigaciones Marinas, Av 8va 1615 Calle 20, Salinas, 241550 Santa Elena, Ecuador; 5 Conservation International, Puerto Ayora, Santa Cruz Island, 200350 Galápagos, Ecuador; 6 Crane Country Day School, 1795 San Leandro Lane, Santa Barbara, CA 93108 USA

**Keywords:** Ecosystem ecology, Marine biology

## Abstract

Throughout the Galápagos, differences in coral reef development and coral population dynamics were evaluated by monitoring populations from 2000–2019, and environmental parameters (sea temperatures, pH, NO_3_^−^, PO_4_^3−^) from 2015–19. The chief goal was to explain apparent coral community differences between the northern (Darwin and Wolf) and southern (Sta. Cruz, Fernandina, San Cristóbal, Española, Isabela) islands. Site coral species richness was highest at Darwin and Wolf. In the three most common coral taxa, a declining North (N)-South (S) trend in colony sizes existed for *Porites lobata* and *Pocillopora* spp., but not for *Pavona  spp*. Frequent coral recruitment was observed in all areas. Algal competition was highest at Darwin, but competition by bioeroding sea urchins and burrowing fauna (polychaete worms, bivalve mollusks) increased from N to S with declining coral skeletal density. A biophysical model suggested strong connectivity among southern islands with weaker connectivity to Wolf and even less to Darwin. Also, strong connectivity was observed between Darwin and Wolf, but from there only intermittently to the south. From prevailing ocean current trajectories, coral larvae from Darwin and Wolf drift primarily towards Malpelo and Cocos Islands, some reaching Costa Rica and Colombia. Mean temperature, pH, and PO_4_^3−^ declined from N to S. Strong thermocline shoaling, especially in the warm season, was observed at most sites. A single environmental factor could not explain the variability in observed coral community characteristics, with minimum temperature, pH and nutrient levels the strongest determinants. Thus, complex environmental determinants combined with larval connectivity patterns may explain why the northern Galápagos Islands (Darwin, Wolf) have higher coral richness and cover and also recover more rapidly than central/southern islands after region-wide disturbances. These northern islands are therefore potentially of critical conservation importance as important reservoirs of regional coral biodiversity and source of larvae.

## Introduction

Recent changes in climate and local environments have turned many coral reefs into highly endangered ecosystems^1^. Physical factors such as temperature-increases and ocean-acidification in combination with compromised ecosystem processes are causes of higher frequencies of mass coral mortality events and subsequent erosion of reef structures^2^; lack of recovery has therefore been widely observed on a global scale. Since predictions for the future of most coral reefs are dire^3^, it is important to understand, at least on regional-scales, the patterns and drivers of reef establishment and persistence as well as degradation and subsequent recovery^4^.

Climate change affects atmospheric dynamics and may amplify the impacts of phenomena of global-scale importance such as the El Niño Southern Oscillation^5–7^ (ENSO). This event results from coupled ocean-atmosphere perturbations^8,9^ and has been implicated, in its warm phase, with regional to world-wide coral mortality events in 1982/3, 1997/8, 2005 and 2015/6^10,11^. However, changes in ocean-atmosphere dynamics may also affect currents and upwelling, with the latter potentially increasing in frequency and magnitude^12^. This may create safer areas for corals due to potential mitigation of ocean warming^13–15^ during increased frequency of severe marine heatwaves^16^. The Galápagos Islands are situated in a region of marginal coral reef development^17^ and are strongly influenced by ENSO as well as variable upwelling dynamics of the Equatorial Undercurrent^15,18–20^ (EUC). The islands experience strong North-South gradients of sea temperatures^17^ and aragonite saturation states^21,22^. There is also a gradient in reef-associated faunal biomass, due to variable fisheries pressure across the archipelago^23^. In general, the Galápagos marine ecosystems are well-protected from anthropogenic impacts and therefore provide an opportunity to gain insights into ecosystem functioning as primarily driven by environmental gradients under large-scale climatic control, rather than degradation driven by local human activities.

The central and southern sectors of the Galápagos Archipelago are cooler and more subject to upwelling influence^14^ compared to the northern islands, Wolf and Darwin (volcanic edifices structurally-independent of the main archipelago^24^) that are more typically tropical^20,25^. Consequently, the biogenic sedimentary dynamics (of which reef growth is a part) differ between these islands. The southern archipelago has more typical cool-water carbonates with a strong heterozoan upwelling overprint^26^, which is less clearly developed in the north^27^. It is also well-known that the northern islands harbor denser scleractinian coral populations and the only remaining framework reef in the entire Galápagos Archipelago^17,25^. Only Darwin and Wolf, the northernmost islands, fall within the classical sedimentological “reef window” with regards to average temperature and nutrient dynamics^26–28^. The gradients in aragonite saturation^21,22,28^, temperature and even the influence of ENSO effects^27^ therefore play strongly into reef dynamics, but it is incompletely understood how the coral (meta)population functions and how connectivity patterns^29,30^ influence coral reef development and reef coral persistence in the Galápagos. A clear difference was observed between coral recovery in the southern islands after the 1982/3 and 1997/8 ENSOs, and those in the northern islands^25^. Thus, differential coral dynamics are evident but quantification of processes is still sparse and literature points to several drivers^4,11,25,27,31,32^.

In this study, we explored the dynamics of coral and reef growth across the Galápagos Archipelago based upon differences in (1) coral size and cover, (2) framework development, (3) coral competitors, predators and bioeroders, (4) larval connectivity patterns based on a biophysical model, (5) correlations of the aforementioned parameters with temperature, pH and nutrient gradients. Of particular interest was the apparent difference in coral size, density, reef-building and recovery dynamics between the northern islands and the rest of the archipelago^18^, and we sought to determine whether these differences are based primarily on physical factors (such as mean seawater temperature, short-term temperature excursions, aragonite saturation state) or if biological dynamics and connectivity alone would suffice to create this gradient.

## Material and Methods

**Figure 1:**
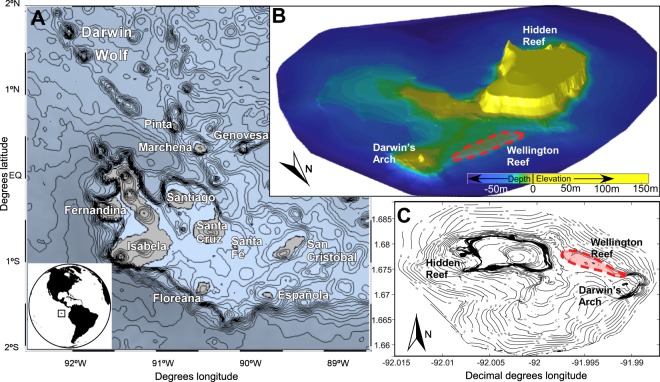
(**A**) Galápagos Islands (Ecuador) study area in the Eastern Tropical Pacific. (**B**) Rendering of bathymetry around Darwin Island, based on 200 kHz single-beam surveys undertaken in this study. Wellington Reef, the only extensive coral framework in the Galápagos, is circled in red. It does not occupy the shallowest part of the Darwin platform. Image is tilted to provide best view of Wellington Reef. Elevations of the island are approximate and not based on a land-survey. (**C**) Contour chart of the same bathymetry as in (**B**). The Hidden Reef is not a framework structure, but an accumulation of relatively large, but isolated coral colonies. Map (**A**) plotted using R package “marmap” and NOAA bathymetry, maps (**B**,**C**) using Matlab and gridding data from present study.

Coral surveys were undertaken across the Galápagos Archipelago (Fig. 1A) from 2000–2019, where regularly monitored transects were revisited in two depth zones (5 and 15 m) at fixed sites. These data were augmented with surveys at additional, haphazardly chosen sites and/or depths, if corals were widely distributed. But corals occur on most islands in relatively small, easily defined areas. Sites visited were those of known occurrences of corals over the past decades^17,25^. Sample sites and data taken at each visit are shown in Table SI-1 (Supplementary information). All analyses were done in the software R^33^. The **occurrence of coral species and their sizes** were evaluated during timed 60-minute swims of comparable distance, when every colony encountered was identified to species and was measured for the horizontal projection of greatest and smallest diameter, as well as vertical extent above the substratum. Additionally, phototransects of 10 m length were taken, each with a graduated meter-long measuring stick at the center to allow easy extraction of coral size and coverage information. Species occurrences were recorded as presence/absence (binary) matrix, on which ordination by Kruskal’s nonmetric multidimensional scaling (in R-package^33^ MASS^34^) was employed using Manhattan distance, which gave the lowest stress value of the variety of binary indices available^35^. PERMANOVA (in R-package vegan^36^) was used to test for significance in patterns of species presence/absence within the entire archipelago. Test-groups were evaluated for homogeneity of multivariate spread^36^, which was rejected. Since this raised the possibility of spurious results, regular ANOVA was also employed to evaluate differences in species richness observed among sampling years and sites. Whenever parametric analyses were used in this study, data were either previously tested or the model (regression or ANOVA) was checked for assumptions of normality and homogeneity of variances using plots of residuals or qq-plots. If necessary, data were appropriately transformed or standardized. In all tests, we considered a minimum significance level of 0.05 as sufficient to reject the null hypotheses.

For analyses of coral growth and **coral sizes** across the archipelago, the most frequent reef-building coral species *Porites lobata* (records likely including the cryptic species *P. evermanni*^37^, which cannot be reliably differentiated in the field) and *Pavona* spp. (pooled across species since they separate in relative importance along depth and among sites, Table SI-2), as well as *Pocillopora* spp. (pooled records, since differentiation of all 9 species from photos or under water was not reliable) were evaluated. Size trends were expressed as contour plots with graduated, extrapolated surfaces of median sizes across the archipelago. Additional information (boxplot) is provided in Supplemental Information (Fig. SI-1). Size distributions in sampling areas were expressed as histograms with 20 cm bin sizes. At three fixed sites at Darwin island, coral sizes and partial mortality were evaluated repeatedly between 2000 and 2018^25,38^. **Partial mortality** was repeatedly observed at the same three sampling sites at Darwin and expressed with logistic regression models on the proportional response data. Density of other benthic biota, such as algae (which became important competitors after 2012), was evaluated by projecting 50 random points onto photographic images (selected by random draw) and noting the identity of the underlying flora/fauna/substratum type. Interference competition, that is direct interaction of corals and algae for the limiting resource of substrate space, was quantified. **Coral competitors and erosion agents** were evaluated by estimating the percent surface area of photographed corals (planar projection surface viewed from above) affected by the respective categories (burrowing bivalves, encrusting cirripeds, grazing sea urchins, macroalgal and turf overgrowths, and fish grazing causing tissue mortality).

To estimate **larval dispersal and connectivity** among known occurrences of reef corals, a biophysical model^39^ simulated the movement of coral larvae (planulae) across a 2-D, fixed cell-dimension grid using stochastic, Lagrangian particle tracking. The grid incorporated the Galápagos and northern Eastern Tropical Pacific (ETP) regions with grid pixel size 1/12^th^ decimal degree (~9 km), using ocean current data from the global HYbrid Current Ocean Model (HYCOM). Daily data from 2005 to 2015 were employed in a ten-year simulation. Planulae diffusion in ocean surface currents and recruitment were tracked from the water column to known coral reefs in the study region. Known coral populations were clustered into precincts that covered the respective coral areas (southern and northern Galápagos, Cocos, Malpelo and Gorgona Islands; La Osa and Guanacaste Peninsulas in Costa Rica, Gulfs of Chiriquí and Panama in Panama, Colombian coast, Ecuadorian coast in Manabí Province). One geographic location every 9 km of known reef occurrence was selected as a site for a reproductively mature coral cluster to seed the simulations (density-independent and one cluster per point, total 350 clusters). Simulations selected reproductive clusters within all precincts at the start of each year. Each mature cluster was allowed to spawn recruits every 30 days from January to May^32^. Larval release was simultaneous from all colonies and randomly staggered within each model colony over five days.

From HYCOM simulations, a daily vectorized flow-field of ocean currents (measured in m/s) was obtained over which planulae dispersed following a Lagrangian path during the pelagic larval duration (PLD, 90 days^39^). The *u* (east-west) and *v* (north-south) positions of the larvae (*P*_*i*_ = east-west, north-south position at time i) and their hourly transition times were tracked. The vector (${\overrightarrow{v}}_{i}$) traversed by any planula per Δ*t* was calculated by bilinearly interpolating the nearest four ocean current vectors to the planula’s *x*/*y* position. A timer tracked hours elapsed over each planula’s PLD. After a pre-competency period of two lapsed days, known coordinates of coral areas within one kilometer of the planula’s position at each Δ*t* were determined. If a planula was within one km of a reef, it was allowed to recruit to that position and then remain non-reproductive for the duration of the simulation (10 years). Planulae were considered lost if they did not come within one kilometer of a suitable recruitment position during their PLD in the models.

Using this protocol, the path from spawning to recruitment was chronicled (as were founder colonies and subsequent recruitment positions) in order to produce a connectivity matrix. To be conservative, since larval survival rates are unknown, only one larva per colony was incorporated in the simulation. Also, modeled larvae were considered representative for all of the most important scleractinian coral species in Galápagos which have similar reproductive strategies (broadcast spawners with similar larval dynamics^32^). A connectivity matrix was developed that cross-plotted the founder clusters on the x-axis with the recruitment positions on the y-axis. Each recruitment position was color coded via a cool (blue - low) to hot (red - high) scale to represent relative densities of recruits. Larval trajectory maps and density maps were also produced of larvae spawned from southern and northern Galápagos populations to illustrate the differing diffusion patterns of larvae from these two regions.

To evaluate large-scale coral **patterns associated with long-term temperature** conditions, we obtained the HadISST 1 × 1 geographic degree temperature tiles^40^ for the Galápagos, which provide a synthetic temperature record from 1870 to the present (from https://coastwatch.pfeg.noaa.gov). To evaluate how reliably this synthetic dataset reflected on-the-ground conditions, we compared it to a 50-plus year dataset of *in situ* temperature recordings that was available from Puerto Ayora^41^. We examined the correlation of mean monthly SST in the time-window 1981–2012 of the synthetic HadISST datasets against that of the Puerto Ayora data. HadISST data correlated highly with the *in situ* data over the comparison interval (linear regression *R*^2^ = 0.88, *p* < 0.05). HadISST data closely tracked locally observed temperature dynamics. However, *in situ* data were lower by ~1 °C, probably a result of the *in situ* thermo-sensor situated in deeper water than the ocean’s surface skin (as considered by SST temperature models) and cool ground water percolating into the area. Extremes in HadISST were of smaller magnitude than *in situ* records. However, the HadISST dataset was considered accurate enough to evaluate the last century and a half of temperature dynamics as a proxy for average coral-growth conditions in the study area.

For evaluation of short-term temperature dynamics, which is known to be highly variable^18,19^, HOBOTempII temperature sensors were deployed at Wolf (10 m, 15 m and 20 m), Darwin (12 m), Fernandina (10 m), Floreana (2 m and 15 m), and Española (15 m), recording *in situ* temperature measurements at 30-minute or hourly intervals. To evaluate patterns in the time series, classical timeseries decomposition was employed, considering the timeseries as additive^42^. First, the trend cycle was calculated using a moving average of twice the cycle-length (2 tidal cycles per day = 24 hours) which was then used to calculate the detrended timeseries. The seasonal component was calculated as the average of all detrended values within a cycle, and the random (remainder) component by subtracting the seasonal and trend-cycle components. Seasonal upwelling intensity (cool period [July to December] versus warm period [January to June]) was evaluated using the random component (thus the non-seasonal, unpredictable or extreme events). When data were non-normal, Wilcoxon tests were used for two-sample comparisons at each site (Darwin, Wolf, Fernandina, Floreana). An upwelling-index was evaluated as the proportion of negative values of all datapoints in the random component.

Surface **water chemistry** samples were collected at geo-referenced locations. Sterile, acid-washed syringes were used to collect water in sterile, acid-washed PVC containers by filtering through disposable hydrophilic PVDF 0.22 micrometer Millipore filters. Water samples were tested with a portable Hach DR900 multiparameter colorimeter for NO_3_^−^ using Hach Nitraver5 reagent pillows, and PO_4_^3−^ using Hach Phosver3 reagent pillows. Temperature and pH were measured with a YSI 556 MPS multiprobe. For evaluation of trends, data were first plotted according to their geographic position, then grouped as suggested by community differentiation, and finally parametrically tested using ANOVA and linear regressions. Samples in the northern islands at Darwin and Wolf were replicated several times daily (whenever possible, three times) annually from March 2016 to March 2019 at the single permitted anchorage site. Samples in the central islands were replicated at the nearest possible location determined by GPS during each visit (Nov. 2016; March 2019). Samples in the southernmost islands were only obtained once (March 2019). All other samples, away from the permitted anchorages, represent unique measurements that were taken while the vessel was under way. They therefore represent random samples.

To visualize the **relative importance of community-intrinsic** (species richness, framework development, number of recruits, median size of coral colonies) **and community-extrinsic** (environmental, sea temperature, upwelling intensity, pH, NO_3_^−^, PO_4_^3−^) **parameters**, we used constrained correspondence Analysis^43,44^ (CCA). A matrix of community data (species richness, median size and number of coral recruits in *Porites lobata*, *Pavona* spp., *Pocillopra* spp, framework height; skeletal density was derived from Manzello *et al*. 2014^22^) was constrained by a matrix of environmental data (connectivity, mean, maximum and minimum temperatures in HadISST tiles, upwelling index, surface concentration of PO_4_^3−^ and NO_3_^−^, size of the area harboring dense coral growth, distance to next coral area, percent of colonies eroded by sea urchins, percent of colonies overgrown by barnacles). Since data within and between the matrices were at different scales, they were standardized to a range of 0–1 by subtracting minima and then dividing by maxima of each variable. An entirely unconstrained model was made to approach a fully constrained model and halted at minimum AIC (Akaike Information Criterion) level^45^. Those constraining variables that had the highest individual AIC-scores, but that in combination as an additive statistical model would lead to the overall lowest model AIC, were retained. These can be interpreted as those environmental variables that best constrained community differentiation.

In 2017 and 2018, a bathymetric survey using a Biosonics MX 200 kHz single-beam echosounder was deployed at Darwin to better visualize the setting of the only framework reef in Galápagos (Wellington Reef^38^). Survey depths were corrected to the mean-tide level over the survey period. To measure tidal change over the survey period, a Hobo Water Level U20L-02 logger was deployed. Gridding and correction of bathymetry in Fig. 1B,C was performed in Matlab.

## Results

### Coral occurrence and species richness across the archipelago

Overall, there are 23–24 scleractinian coral species recorded from the Galápagos^46^, 22 of which were encountered in the present surveys. The main reef framework builders belonged to three genera: *Porites lobata*, *Pavona clavus* and *Pocillopora* spp. No well-developed *Pocillopora* frameworks are presently known, these having been eroded after mortality during the 1982/3 and 1997/8 ENSO events^47^. An aggregation of *Pocillopora* colonies at Isabela (Concha de Perla lagoon^48^) were coalescing in April 2012, though a reef framework structure had not yet formed. Unsurprisingly, due to the much denser nature of coral populations at Darwin and Wolf, more species were encountered at those two islands than at any other site during our surveys (Table SI-2). Sites on Fernandina had the lowest species richness (Table SI-2), with only one species (*P. gigantea* at Pta. Espinosa), followed by the W-coast of Isabela (*P. gigantea*, *P. lobata*), Santa Fé with three, and the E-coast of Isabela with four species (Table SI-2). Some species had only very local distributions. *Leptoseris scabra* and a yet unidentified *Leptoseris* sp. 1 (Glynn PW, pers. comm.) have only been encountered at Darwin and Wolf. *Diaseris distorta* is currently known from several extensive assemblages at Floreana near Corona del Diablo Island^49^, although dead skeletons were observed near Gardner Bay, Española. *Cycloseris curvata* was common at Corona del Diablo (Floreana) in the 1970s through 1992, but has almost completely disappeared recently (2000–2019). Also, *C. curvata* was once present at Española and Darwin, but only dead skeletons of unknown age were encountered at these sites. Across all surveys, significantly more species (summed species occurrences at each island) were recorded at Darwin and Wolf than at all other sites (ANOVA, *F* = 80.4, *df*: 11,25, *p* < 0.001; differences between Darwin and all islands, except Wolf, highly significant, *p* < 0.001; see Table SI-2 for listing of species, Table SI-3 for the ANOVA table).

**Figure 2:**
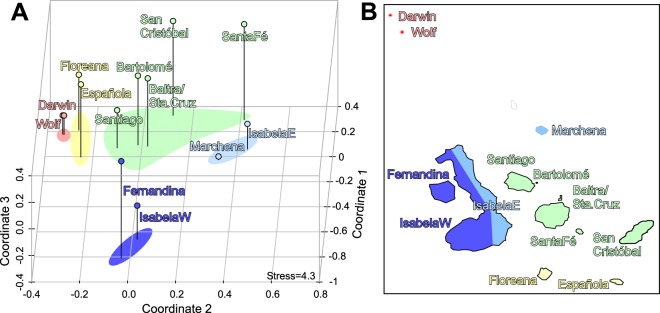
Differences in assemblages of scleractinian coral species based on presence/absence. **(A)** Multidimensional scaling based on city-Block (Manhattan) distance between sites (all individual sites were pooled for each island). **(B)** Statistical groupings of coral species assemblages shown in (A) superimposed as color-code on map of the Galápagos islands.

An nMDS evaluated the matrix of between-site similarity of species presence/absence based on Manhattan distance, expressed in three dimensions (Fig. 2A). Addition of the third dimension significantly reduced stress values (from 9.8 in 2-D ordination to 4.3 in 3-D ordination). The analysis clearly separated four groups of islands according to distances between sites with regards to species occurrences and richness, following approximately the major geographic and oceanographic divisions in the archipelago (Fig. 2B). The identified groups consisted of the northern islands (Darwin, Wolf) with the greatest species richness, the southernmost islands (Floreana, Española) with the second highest species richness, the central islands (Santiago, Bartolomé, Baltra/Sta. Cruz, Santa Fe) and as a separate, more depauperate group, Marchena and the eastern shoreline of Isabela. The western islands (Fernandina and western shoreline of Isabela) clearly separated with the lowest species richness (only *P. lobata* and *P. gigantea*). These groups differed in multivariate spread (ANOVA on betadispersion, *df*: 4,8; *F* = 3.9; *p* = 0.046) but a PERMANOVA detected significant differences in means in the multivariate dataset of species richness based on the occurrences of individual species (*F* = 5.58; *df*: 1,11; *p* = 0.001).

Species richness at each island did not correlate with island area as proxy of coastline and shelf area available for coral growth (*R*^2^ = 0.08), but it increased with the estimated area actually covered by appreciable coral growth (=area in which >1 coral colony occurred in 100 m^2^; y = 54.5x + 7.11; *R*^2^ = 0.49, *p* = 0.01). This area significantly increased from the southern to the northern islands (ANOVA between groups defined by MDS above, *F* = 5.38, *df*: 1,10, *p* = 0.043), being greatest at Darwin (the second-smallest sampled island), where most of the island’s subtidal substrate supports at least some coral cover (Fig. 1).

### Trends in coral colony sizes across the archipelago

**Figure 3:**
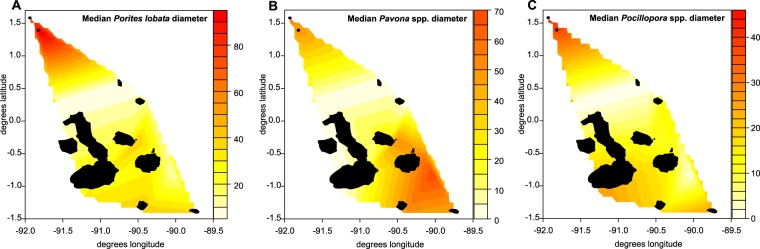
Interpolation surfaces showing colony diameter size trends in the most common coral genera across the sampled area in the Galápagos. Note differences in respective scale sizes. In (**A**,**C**) a bimodal tendency of declining coral size from N and S towards the center of the archipelago is observed. The boxplots on which this figure is based are shown in the Supplemental Information Fig. SI-1. No size class information from San Cristobal was available, hence this region is not included in the prediction surface.

**Figure 4:**
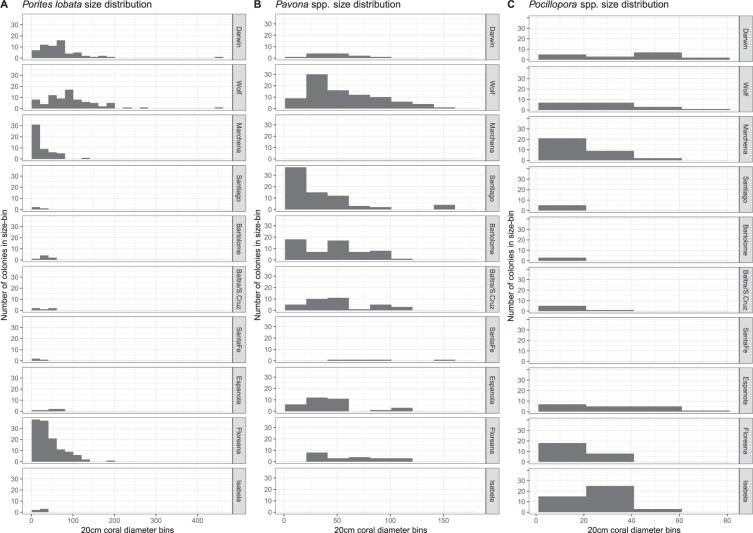
Coral colony frequency of occurrence in 20 cm diameter size bins across all Galápagos survey sites.

Reef-building corals occurred throughout the entire Galápagos in greater or lesser density and formed assemblages with subtle differences (Fig. 2). Darwin Island was the locus of the only extensive reef framework, known as Wellington Reef^46^, that was continuous over hundreds of meters and home to some of the largest coral colonies. Corals at Darwin and Wolf were clearly larger than at all other sites (Fig. 3, Fig. SI-1). Highest median *Porites lobata* colony size was found at Wolf, followed by Darwin, which is attributed to a large proportion of small, densely positioned colonies. The most frequently and consistently encountered coral was *P. lobata*. *Pavona* spp. exhibited the highest median size at Darwin, but large colonies also occurred at the southern islands (Fig. 3A,B). Only dead *P. clavus* colonies were encountered during surveys at Marchena and Sta. Fé. *Pocillopora* colonies grew to largest size (greatest median diameter) at Darwin and Wolf (where the largest colonies occurred overall), but were also notably large at the southern sites at Española (near Gardner Island). *Pocillopora* species were not encountered at the Santa Fé, Fernandina or western Isabela sampling sites. At eastern Isabela, an incipient *Pocillopora* framework of ~1650 colonies was present at Concha de Perla Lagoon. Colonies up to 75 cm in diameter were encountered in 2012 but mortality reduced the largest colonies to 43 cm diameter by 2015 (Fig. 4C).

Figure 3 shows a gradient in colony sizes from the northern islands (Darwin, Wolf) to the main archipelago in *Porites lobata* and *Pocillopora* spp. Differences between islands were highly significant in *P. lobata* (ANOVA of log-transformed data: *F* = 14.72, *df*: 9,338, *p* < 0.001; colonies at Wolf were significantly larger than at all other sites; details in Table SI-4). At Española a few colonies with larger variance resulted in non-significant differences although mean sizes were clearly smaller. Also, in *Pocillopora* size differences between islands were highly significant (ANOVA: *F* = 5.3, *df*: 8,163, *p* < 0.001; colonies at Darwin and Wolf were significantly larger than at all other sites, Table SI-5). In *Pavona* spp. the size-gradient was less clear (ANOVA overall significant, *F* = 8.3, *df*: 7,317, *p* < 0.001; but pairwise differences among sites were significantly different only between Darwin, Santiago and Bartolomé, Table SI-6). Pooled across all three genera, corals at the northern islands of Darwin and Wolf demonstrated significantly larger mean diameters than those in the southern archipelago (ANOVA, *F* = 7.3, *df: 9,165, p* ≪ 0.001).

Coral size-class frequency distributions across the archipelago revealed presence of the smallest size class at most sampling sites, which is indicative of ongoing sexual recruitment. The measured corals (*P. lobata*, *Pavona*, *Pocillopora*) showed a preponderance of middle size classes, suggesting irregular recruitment. In some areas and species, a second mode in the very large size-classes was observed (Fig. 4). Overall, the largest corals were *P. lobata* at Wolf and Darwin, reaching >4 m in diameter (Fig. 4A). The largest *P. clavus* colonies were present at Wolf, but the size-distributions in this genus showed almost equally large, but not as many, colonies in the central and southern islands (Fig. 4B). *Pocillopora* spp. colonies were largest in the northern islands with a few large colonies also present at Española (Fig. 4C). Numerous large colonies of *P. lobata* and *P. clavus* were present in the southern islands in the 1970s, several over 200 yr in age^50^, p.104, but no longer observed in recent surveys (at least since 2012).

**Figure 5:**
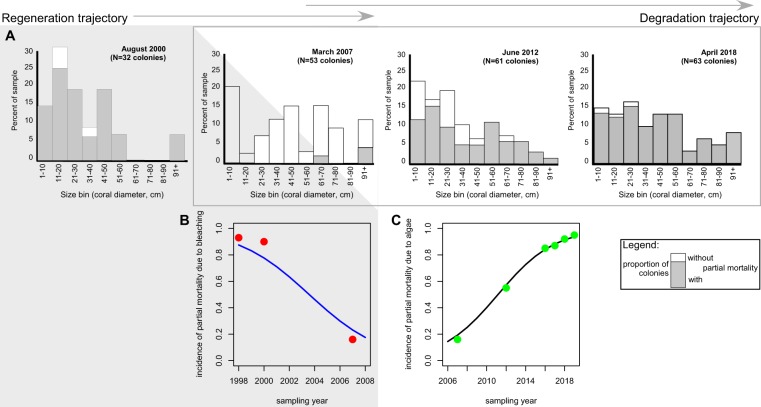
Regeneration versus degradation dynamics as observed at Darwin’s Wellington reef in response to bleaching mortality in response to the 1998 ENSO and an increasing algae invasion beginning shortly after 2007. (**A**) Upper row: Size distribution of linear skeletal growth axes (approximately the greatest vertical height of colony) of *Porites lobata* in four monitoring periods at three monitoring sites. Gray section of bars in (**A**) denote proportion of colonies with dead patches. Data from 2000, 2007 and 2012 from refs^25,31^. (**B**,**C**) Lower row: Logistic regression analyses show the decline in partial mortality that had occurred from bleaching with increasing time after the event, and partial mortality increase in response to increased algal overgrowth.

The coral size distributions among sites were not uniform, suggesting site-specific differences in recruitment and growth patterns across the archipelago. Size distributions are reflective of both recruitment and partial or whole-colony mortality in response to predation, bleaching and/or diseases. At three fixed sites at Darwin, *P. lobata* size distributions were sampled since 2000^25,38^. Smaller size-classes were prominent, but these were not the most frequent in all years. Colony size distributions at the Wellington Reef sites were different (Fig. 5A) than those measured across all sites at Darwin (including Hidden Reef and non-reef sites, Fig. 4A). From 2007–2018, the ratio of large colonies increased, while that of the smallest size-class decreased – sign of an aging population. In 2000, most sampled colonies exhibited partial mortality due to the 1998 coral bleaching event. In 2007, most colonies had recovered (Fig. 5B) and few signs of partial mortality were observed (Fig. 5B; logistic regression from the quasibinomial general linear model showed the trend but was not significant, likely due to low *N*; *t* = 2.195, *p* = 0.116; null deviance: 320.1, *df:* 4, residual deviance 43.8, *df*: 3). Frequency of partial mortality increased over the next 10 years. By 2017, almost all colonies showed partial mortality, which coincided with increasing invasion by the algae *Caulerpa racemosa* and *Caulerpa chemnitziae*. By 2017, these were overgrowing many surfaces of *P. lobata* colonies of all sizes (Fig. 5C; logistic regression quasibinomial GLM showed a significant relationship, *t* = 25.43, *p* < 0.001, null deviance: 443.1, *df*: 7, residual deviance 2.53, *df*: 6). Thus, while the incidence of partial mortality first declined with distance to the last severe bleaching event (2000–2007, from ~90% to ~10%, “regeneration trajectory” in grey in Fig. 5A,B), it increased with the invasion of an aggressive competitor (2012–2018, from ~50% to ~95%; “degradation trajectory” in Fig. 5A,C).

**Figure 6:**
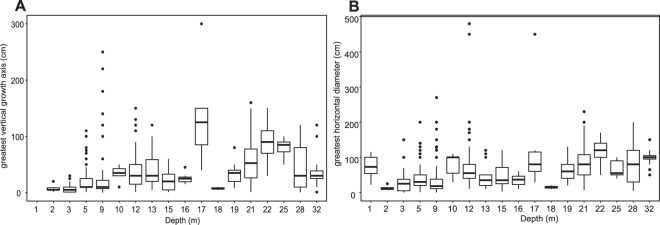
Depth distribution of coral sizes (all species, all island locations) measured as (**A**) vertical growth axis (**B**) maximum horizontal diameter. Note that x-axis is not regularly continuous, but shows depths at which samples existed.

Unlike the clear horizontal differentiation of coral colony sizes along latitude, no clear vertical pattern with depth was observed. Smaller coral colonies were distributed across the entire sampled depth-range (0–35 m) but were most common at 0–5 m. As clear outliers, the largest corals were present in a depth-range between 9 and 17 m; this was most pronounced at Wolf and Darwin (Fig. 6). When coral diameters and heights were binned into five-meter depth-intervals, rank-based testing revealed significant differences across depth (Kruskal-Wallis, for coral diameter: *X*^2^ = 110,1, *df*: 7, *p* < 0.001; for coral height: *X*^2^ = 138,9, *df*: 7, *p* < 0.001). Corals in the first five meters were generally smaller than those at greater depth (Fig. 6). An Analysis of Covariance (ANCOVA) assuming depth (0–32 m) and site (=12 islands) as independent factors, with coral diameter as the variable, suggested that site had a significant influence (*F* = 18,9, *df*: 1,9, *p* < 0.001) while depth was non-significant (*F* = 3.57, *df*: 1,9, *p* = 0.059; Table SI-7). Assumption of an interaction between site and depth in its influence on coral size was highly significant (*F* = 3.45, *df*: 9, *p* = 0.0004; Table SI-8). Thus, the setting of each island influences the depth distribution of corals, which differs primarily among northern islands versus southern islands. The northern islands exhibit greater ecological and/or morphological differentiation along the depth gradient that influences coral growth, and thus size.

### Coral frameworks

Laterally extensive framework reefs are restricted to Wellington Reef on Darwin Island (Fig. 1B,C). Some small, incipient frameworks also exist at Floreana (Tres Cuevitas and Corona del Diablo^49^), at Wolf (Bahía Tiburón), and at Concha de Perla lagoon at Isabela (Table 1). Prior to the 1982/3 ENSO, more framework reefs existed in the southern islands, but these have since been eroded^50^. Wellington Reef is situated between Darwin’s Arch and the island within the depth-zone (9–17 m) of observed largest corals (Figs 1 and 6) where an erosional platform in tuffaceous strata was planed to below sealevel (Darwin’s Arch itself also consists of tufa and pyroclastic layers). Elsewhere on Darwin, corals occurred all around the island, but did not develop frameworks and were widely spaced along the steep depth gradient, comparable to Wolf. Apart from the above-mentioned areas, corals occurred in sparse or even relatively dense associations throughout the archipelago, as individual colonies growing on a non-limestone substratum. Small incipient frameworks were observed only in a few areas (Table 1).

**Table 1:** Reef framework thicknesses in Galápagos between 2000–2019.

Location	Main framework builder	Framework type	Morphology generating	Sediment retaining	Framework thickness
Darwin	*Porites lobata*	True framework, lateral extent >100 m	Yes	Yes	>4 m
Wolf	*Porites lobata*	Incipient, lateral extent <20 m,	Yes	No	Max. 4 m
Floreana, Corona del Diablo^65^	*Porites lobata*	Incipient, lateral extent, ~10 m,	Yes	No	1 m
Floreana – Tres Cuevitas	*Pavona clavus*	Incipient, but most colonies not fused	Yes	No	<1 m
Santiago and Bartolomé	*Pavona clavus*	Incipient, but most colonies not fused	Yes	No	<1 m
Isabela-Concha de Perla lagoon	*Pocillopora damicornis*	Incipient, some fusion of colonies	Yes	Partly	<1 m

### Competitors and predators of coral

**Figure 7:**
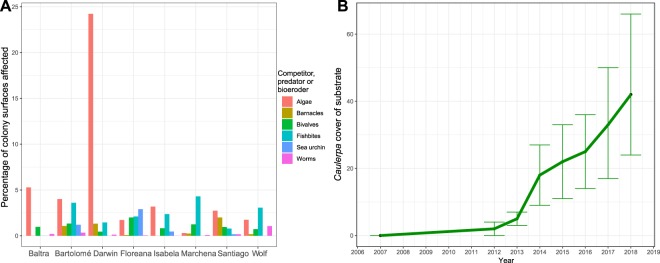
Coral competitors and signs of predation. (**A**) Shows the incidence of interaction with corals by competitors, predators and bioeroders across the Galápagos archipelago. Y-axis represents the percentage of coral colonies affected. (**B**) Incidence of *Caulerpa* spp. in phototransects (*N* = 160 images, datapoints are means +/− S.D.) at Darwin. Y-axis represents percent cover on the reef framework. Some corals were overgrown, but the algae primarily covered coral rock.

Corals were frequently overgrown or eroded by other organisms (Fig. 7A). Macro- or microfilamentous algae were commonly observed competitors, with the overall highest incidence at Darwin, where also cirripeds were found in great abundance. Visible influence of predators was evident throughout the archipelago, with fish bite-marks peaking at Marchena and Wolf. Signs of sea urchin grazing/abrasion were very clear in the southern islands, especially at Floreana (Tres Cuevitas). Significant differences between the islands were found in the proportion of coral surfaces affected (Kruskal-Wallis tests due to non-normality of residuals, all with *df*: 7) by barnacles (*X*^2^ = 102.8, *p* < 0.001), turf algae (*X*^2^ = 38.9, *p* < 0.001), burrowing polychaete worms (*X*^2^ = 108.28, *p* < 0.001), sea urchins (*X*^2^ = 89.4, *p* < 0.01), fish bite marks (*X*^2^ = 0.191, *p* < 0.001) and burrowing bivalves (*X*^2^ = 37.24, *p* = 0.001). Also, a PERMANOVA detected overall significant differences in means in the multivariate dataset based on the surface percentage of coral affected by these categories (*F* = 11.05; *df*: 1,216; *p* = 0.001). However, multivariate spread among the groups was not homogeneous (ANOVA, *F* = 4.68, *p* < 0.001), which could have influenced the outcome.

Algae were observed in direct competition with corals at many sites (Fig. 7) but the abundance of dead *P. lobata* coral framework at Darwin served as a particularly suitable foothold from which to overgrow corals. In the southern islands, turf algae primarily colonized regions on corals freshly devoid of tissues or with moribund tissues (caused, for example, by fish predation), but generally encroached less on live, healthy tissues. At Darwin from 2007–2012, *Peysonnellia boergesenii* overgrew substratum and actively colonized corals, leading to partial mortality. From 2012 onwards, at least two *Caulerpa* species and *Asparagopsis taxiformis* began to rapidly occupy non-coral substratum and impinge on corals. As of 2016, overgrowth competition with *P. lobata* was frequent, leading to partial coral mortality (Figs 5 and 7). In 2017 and 2018, *Caulerpa racemosa* and *C. chemnitziae* carpeted most free substratum between coral colonies over wide areas (~40% of the sampled area) of Wellington Reef (Fig. 7B). At Wolf, this situation had not occurred and only individual tufts of *Caulerpa* had yet been encountered, leaving *P. boergensenii* as the major algal competitor for corals until 2018.

### Larval connectivity within the Galápagos

**Figure 8:**
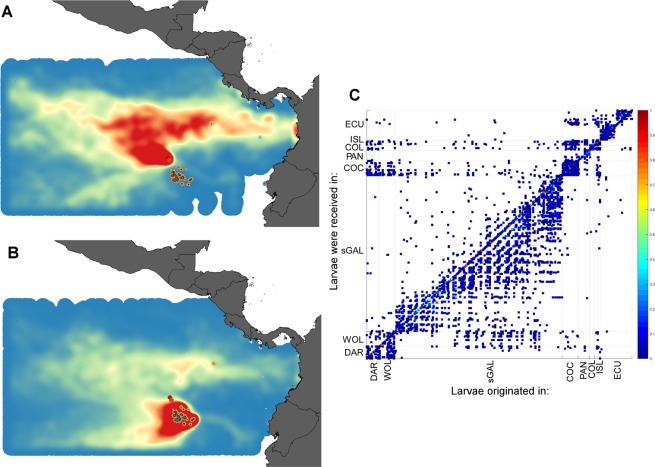
Biophysical model of larval connectivity in the Galápagos and the Equatorial Eastern Pacific, model region with source and settlement areas. The Galápagos Islands and other well-known coral areas in Costa Rica (Cocos Island), Panama (Gulf of Chiriquí), Colombia (Malpelo and Gorgona Islands) and Ecuador (Isla la Plata) are included in this analysis (**A**) Expected settlement density of larvae released from Darwin and Wolf. Red is highest density, blue is lowest (**B**) Expected settlement density of larvae released from the southern archipelago. Red is highest, blue is lowest density, white was not reached by any larvae and/or is outside of the model’s domain. (**C**) connectivity matrix. X-axis represents release sites, y-axis represents “recruitment to” sites. If dots fall within any of the vertical and horizontal boxes with the same name, self-recruitment occurred (i.e. dot falls into sGAL on x-axis but COC on y-axis indicates larva from south Galápagos settled at Cocos Island). Strong connectivity within the southern Galápagos, and sparser recruitment from northern islands into southern is evident. (DAR = Darwin, WOL = Wolf, sGAL = southern Galápagos, COC = Cocos Island, PAN = Panama, COL = Colombia, ISL = Isla la Plata, ECU = Ecuador).

Larval connectivity was evaluated from a model analysis only. While coral recruits were observed and counted (Fig. 4), their provenance could not be established. A biophysical connectivity model simulated pathways of larval transport within the Galápagos and adjacent ETP areas. The model suggested sites within the southern archipelago to be well-connected (most islands experienced exchange of larvae, Fig. 8). It further suggested Darwin and Wolf to receive larvae from the south, but contributing few larvae towards the south in return (Fig. 8). Most larval trajectories originating in the northern islands were directed away from the Galápagos either towards the central Pacific or Central America and Colombia. This resulted in weak two-way connectivity of southern populations with the abundant coral communities at Darwin and Wolf. Galápagos connectivity to the mainland coast (i.e. receiving larvae from mainland Ecuador and Colombia) was intermittent and not very strong (Fig. 8C), but occurring mainly in the warm, coral reproductive season (January-April) when the Panama Jet is active. Together with Darwin and Wolf, the entire Galápagos appeared an important source area for other ETP locations, especially Cocos, Malpelo and Gorgona islands. But Galápagos larvae originating from the northern islands in the model also reached Meso-America (with the possible exception of the Gulf of Panama), Colombia and mainland Ecuador (Fig. 8A,B). Corals in the Galápagos seemed to form a metapopulation that favors the northern islands. Connectivity patterns alone provided strong support to explain the abundant coral populations in the northern-most reaches of the archipelago.

Larval trajectories were evaluated during ENSO and non-ENSO events. Patterns of larval dispersal in the Galapagos archipelago during ENSO years (2005, 2010) were similar with regards to south-north dispersal (Fig. SI-2).

### Temperature as a driver of coral dynamics

Long-term (1870–2018) monthly mean sea temperatures (HadISST) showed a clear gradient in average temperature from the warmer northern islands (Darwin mean SST: 26.28 °C, range: 23.82–30.26 °C) to the cooler western and southern part of the archipelago (Isabela mean SST: 23.83 °C, range: 19.34–30.11 °C; Fig. SI-4). There was no significant statistical relationship between average overall (across all species) coral colony diameter with mean, median, or minimum temperatures in the HadISST tiles (linear regression models: mean sizes against minimum temperatures: y = 68.12–1.322x, *R*^2^ = 0.22, *p* = 0.79; mean sizes against long-term temperature mean: y = 102.6–2.53, *R*^2^ = 0.22, *p* = 0.77; mean sizes against maximum temperatures: y = 728.5–24.6x, *R*^2^ = 0.03, *p* = 0.74). For each species or species complex evaluated separately (*P. lobata, P. clavus, Pocillopora* spp.), some significant linear relationships existed between mean colony size and either mean temperature (*P. lobata*: y = −647.5 + 28.2x, *R*^2^ = 0.81, *p* = 0.009; *P. clavus*: y = 169.39 + 8.76x, *R*^2^ = 0.13, *p* = 0.48, *Pocillopora* spp.: y = −275.6 + 12.03, *R*^2^ = 0.67, *p* = 0.03), minimum temperature (*P. lobata*: y = −200.7 + 16.5, *R*^2^ = 0.84, *p* = 0.01; *P. clavus*: y = −57.64 + 4.95x, *R*^2^ = 0.12, *p* = 0.49, *Pocillopora* spp.: y = −128.7 + 7.13x, *R*^2^ = 0.74, p = 0.02) or maximum temperature (*P. lobata*: y = −5516.1 + 184.5x, *R*^2^ = 0.49, *p* = 0.11; *P.clavus*: y = −1478 + 50.58x, *R*^2^ = 0.05, *p* = 0.64, *Pocillopora* spp: y = −2435 + 81.5x, *R*^2^ = 0.46, *p* = 0.14). The influence of mean and minimum temperatures on *P. lobata* and *Pocillopora* spp. colony sizes, and the absence of any influence on *P. clavus*, is supported by the size gradients throughout the archipelago (Fig. 3). While temperature seemed to be an important driver in some species, history, local conditions and other physical factors may also have played an important role in shaping the composition of coral communitites.

**Figure 9:**
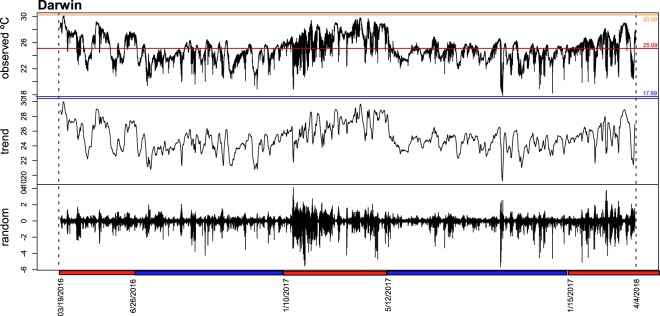
Time series decomposition of water temperatures (half-hourly) on Wellington Reef at Darwin Island (13 m depth) from 03/19/2016 to 4/4/2018. Upper graph: recorded temperatures. Middle graph: 7-point running mean as indicator of trend, Lower graph: Anomalies (deviation from overall mean). Strong downward spikes are the random component (time series – seasonal trend – trend cycle) and thus indicate events that occur irregularly (or at least not determined by the seasonal trend or cycle), such as thermocline shoaling (upwelling). The decomposition assumes a daily period (48 measurements per period; graph not shown since visually too dense). Blue bar = cool seasonal period, red bar = warm seasonal period.

Coral health is often determined by pulses of extreme heat or cold that may be masked by monthly mean temperatures. Half-hourly time series over two years at Darwin showed a similar mean temperatures as the monthly records (Fig. 9), but demonstrated fluctuations of up to 9 °C within 24 hours. A warm season in which daily temperatures were mostly above the annual mean existed from January–May/June, and a cool season with daily temperatures mostly below the annual mean. This pattern was punctuated by strong downward spikes that were more frequent than strong upward excursions and occurred at Darwin more often during the warm than cold seasons (blue and red bars, bottom of Fig. 9), confirming the upwelling influence in the area. Also, the warm period during the 2015/6 ENSO lasted into June, while in 2017 it ended, more typically, in May (Fig. 9).

Hourly temperature records at three sites with declining coral cover and richness (Bahía Tiburón site, Wolf, with developed frameworks; Corona del Diablo site, Floreana, with incipient framework; and Cabo Douglas site, Fernandina, with very few, scattered colonies only) showed a gradient in frequency and severity of thermocline shoaling with rapid cooling, the events increasing from Wolf to Floreana to Fernandina during the same time-interval (Fig. SI-5). At Wolf, temperature declines of about 5 °C in the space of 4 hours were recorded, while at Floreana and Fernandina the range of temperature drops was up to 8 °C. Fernandina was overall the coldest site. Effects of the 2015/6 ENSO event were seen at Wolf, with mean temperatures in 2015/6 elevated by 1.83 °C in comparison to Darwin in the following year 2017 (Fig. SI-5; usually, annual mean temperatures at Darwin and Wolf are similar). At Fernandina and Wolf there were no significant differences in the random component of temperature excursions between the warm (January–June) and colder seasons (July–December; Wilcoxon tests; Fernandina *W* = 6542900, *p* = 0.204; Wolf: *W* = 9492600, *p* = 0.106), suggesting that strong temperature drops occurred at any time in any season. However, at Floreana significant differences were observed during 2015/16 (*W* = 23848000, *p* < 0.001), as at Darwin in 2017 (*W* = 39542000, *p* = 0.047). These contradictory results indicated high variability in upwelling dynamics. Of all measured areas, Fernandina had the highest upwelling index (0.49), followed by Wolf (0.44) and Floreana (0.42), while Darwin had by far the lowest upwelling index (0.19) during the sampled periods.

### Nutrients and pH as drivers of coral dynamics

**Figure 10:**
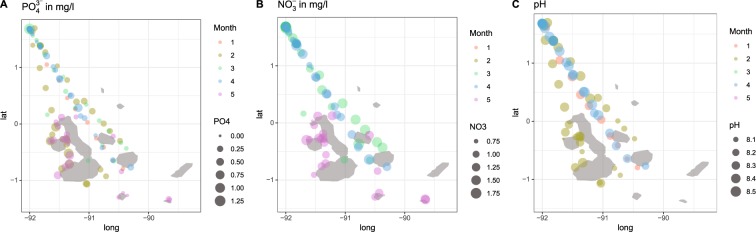
Phosphate (**A**), Nitrate (**B**), and pH (**C**) measurements across the Galápagos Archipelago in March 2016, 2018 and 2019 (“Months 1, 3, 4”), during the warm seasons, and November 2016 (“Month 2”) at the end of the cool season (compare with Fig. 9). Nutrient measurements in mg.l^−1^.

Nutrients (NO_3_^−^ and PO_4_^3−^) measured in March 2016, 2018, 2019 and November 2016 were grouped according to regional oceanographic characteristics for testing (western region = Isabela and Fernandina; northern region = Darwin and Wolf; central region = all other islands; Fig. 10). Nutrient values were generally higher in the central islands than at Darwin and Wolf (ANOVA NO_3_^−^, *F* = 3.98, *df*: 1,76, *p* = 0.05; PO_4_^3−^: *F* = 22.16, *df*: 1,129, *p* < 0.001). However, at both northern islands (Darwin and Wolf) nutrient levels were elevated with respect to surrounding ocean waters, but the relationship was only significant for NO_3_^−^ (Fig. 9; ANOVA Wolf-Darwin-Ocean, NO_3_^−^, *F* = 4.11, *df*: 1,76, *p* = 0.05; PO_4_^3−^: *F* = 1.32, *df*: 1,129, *p* < 0.25). Elevated nutrient concentrations at these islands coincided with strong thermocline shoaling at the time of nutrient measurements (Fig. 10), and likely reflected the advection of nutrient-rich, sub-thermocline waters. Both in the warm and cool seasons, the northern islands are surrounded by generally nutrient-poorer waters than the central and western islands (Fig. 10). The higher nutrient levels at Darwin and Wolf than in nearby open oceanic waters, suggest local influence exerted by the islands upon thermocline shoaling (topographic steering, possibly aided by canyons leading from the island shelf towards deeper water, Fig. 1). Spot-measured pH was generally higher near the northern islands but showed high variability, depending on the status of thermocline shoaling at the time of measurement. During observed upwelling at Darwin, lower pH values, comparable to the southern islands, were recorded. Across all measurements, pH was significantly lower in the central and western islands (ANOVA, *F* = 4.32, *df*: 1, 72, *p* = 0.04).

### Multivariate coral community relationships with environmental variables

Strong univariate relationships existed between environmental and biological factors with species richness and framebuilding across the archipelago (as suggested by Fig. 2). Since significant differences among the island groups occurred in several variables (see Fig. SI-6), further analysis was required to better understand the interactions and the ranking of these abiotic factors as probable determinants of coral community and reef-building dynamics. Canonical analyses, such as CCA, are suitable to constrain the relationships of biological groupings with environmental variables and to demonstrate their relative importance.

**Figure 11:**
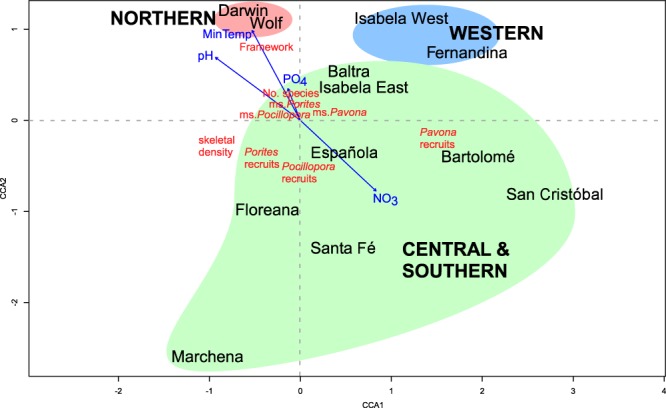
Correspondence analysis triplot showing site separation along first two correspondence axes (CAs) between sites, biological and environmental determinants of clustering. The same colors as in Fig. 2 are used for showing groupings. Major groups of sites separate around the centroid (0, 0; origin of blue arrows)– clearly separating the northern, the western and the remaining central and southern islands. Text in red denotes the community characteristics that structure the ordination of the sites. The community biplot (community characters by sites) scales as community characteristics (red) explaining the site-pattern (scaling 1^43^). Blue arrows are environmental variables used heuristically to explain the variation in community characteristics at sites (making this a triplot). (Abbreviations: CCA = canonical axis, in red: *Pavona* recruits = N *Pavona* recruits, *Pocillopora* recruits = N *Pocillopora* recruits, *Porites* recruits = N *Porites* recruits, skeletal density = skeletal density, med.size_Por = median size of *Porites* colonies, med.size_Poc = median size of *Pocillopora* colonies, ms.Pavona = median size of *Pavona* colonies, Number species = number of species; in blue: MinTemp = minimum HadISST temperature, NO_3_^−^= field-measured concentration in mg.l^−1^ at surface, PO_4_^3^^−^ = field-measured concentration in mg.l^−1^ at surface).

Stepwise model building employed AIC of individual constraining variables to achieve an optimally constraining multivariate model. This analysis demonstrated that the most significant environmental variables determining coral community structure and framebuilding were pH (*AIC* = 6.38, *F* = 2.78, *p* = 0.035) and long-term minimum temperatures (as obtained from HadISST data; *AIC* = 5.01, *F* = 2.9, *p* = 0.20), followed by PO_4_^3−^ levels (*AIC* = 3.53, *F* = 2.63, *p* = 0.030) and NO_3_^-^ levels (*AIC* = 3.23, *F* = 1.47, *p* = 0.18; NO_3_^−^ was no longer significant). Sites separated along similar clusters as in the nMDS (Fig. 2) with groupings consisting of the northern islands Darwin and Wolf, the western islands (Isabela West and East, Fernandina and also Baltra), and the central and southern islands. The southern islands (Floreana and Española) did not form an independent cluster in this analysis. The most influential coral characteristics (those closest to the centroid) separating the groups along this axis were (a) the number of species per site (“No.species” in Fig. 11), (b) median sizes of *P. lobata* (“ms.*Porites*”) and *Pocillopora* (“ms.*Pocillopora*”), as well as (c) thickness of frameworks (“Framework” in Fig. 11). Another perpendicular split was observed (a) along scores of median colony size of *P. clavus* (“ms.*Pavona*”), (b) recruit frequency of *P. lobata* and *Pocillopora* (“*Porites* recruits”, “*Pocillopora* recruits”) and (c) skeletal density.

## Discussion

This study confirms and clearly outlines the striking differences in coral dynamics and reef growth across the Galápagos Islands, as already mentioned by preceding studies^17,18,21,25,47^. The archipelago is divided into a more tropical domain at Darwin and Wolf^51^ that contains large and vibrant corals with clear signs of frequent recruitment, and a cooler domain in the southern islands that generally contains smaller and presumably younger coral colonies (in *Porites* and *Pocillopora*, but not in *Pavona*), fewer recruits, and is presently lacking reef framework development. The general pattern of more tropical northern and more temperate southern assemblages not only holds true for scleractinian corals but also for foraminifera^28^, and carbonate sediments in general^26–28^. From a perspective of sea temperature and nutrients, only the northern islands Darwin and Wolf fall within the sedimentological “reef window”^26,27,52^ – and this is clearly borne out by the scleractinian coral fauna, which reaches maximum diversity and space cover in the northern domain^18^.

### Coral occurrence across the archipelago and connectivity

There is noticeable differentiation in scleractinian (reef-building coral) fauna across the islands. While many species occur throughout the archipelago and beyond in the ETP, not all species are found at all sites and a marked gradient of species-richness is observed from the western to the southern and to the northern islands. This pattern is supported and explained by the connectivity patterns in the biophysical model, which suggests that the southern archipelago is well-connected throughout and that the northern islands receive larvae from the south (Fig. 8). Although Darwin and Wolf have a more typically warm-tropical environment that supports greater coral diversity than the rest of the Galápagos, the possibility of achieving even higher levels of diversity may be limited by the cooler upstream source for larvae. If a richer coral species diversity were to develop at Darwin or Wolf, the limited N-S connectivity would make it unlikely for this diversity to be exported to the southern islands. Similarly, the northern islands have more limited potential to serve as a direct refuge for southern coral populations in case of the latter’s decimation by impacts differing between these environments. Larval connectivity pathways shown by our model suggest that southern islands would receive just enough larvae from Wolf to allow for limited, probably very slow, recovery, as was indeed observed^47^. Genetic entities that originated from the northern islands may return to the southern archipelago via migration along the northwestern South American coast, where larvae from Darwin and Wolf may have settled^29,30,53^. During activity of the Panama Jet, which occurs around the start of the coral reproductive period in January^32^ connectivity is established from the South American mainland to the Galápagos^53^ (Fig. 8). Genetic studies suggest that at least in *P. lobata*, gene-flow is high within the ETP^53,54,55^ which is supported by our findings.

The observed patterns of connectivity help explain why coral recovery following the 1982/3 and 1997/8 ENSO-mediated mortality was much slower (and absent in some areas) in the southern islands, where the bioerosion of coral frameworks was more pronounced^56,57^, while rapid recovery occurred at Darwin and Wolf^4,25,38^. The much greater isolation of the southern archipelago from upstream larval sources puts it at a disadvantage with respect to larval recruitment in comparison to Darwin and Wolf, which receive larvae from the south and also benefit from significant self-recruitment from local sources. While some corals spread their gametes far and wide, others may be very local in the dispersal of their reproductive products, like the dense assemblage of *Pocillopora* that formed asexually at Isabela (Concha de Perla lagoon, near Puerto Villamil)^48^. Conceivably many other such assemblages, and perhaps even reef frameworks, may have originated by asexual propagation. For example, a *Pocillopora* framework existed at Punta Espinosa (Fernandina) but disappeared after the 1982/3 ENSO^17,47^ and only a few corals (*Pavona gigantea*) have been observed there in recent surveys. Such local coral populations, especially if asexually-produced, would be difficult to replace after region-wide mortality (1982/3, 1997/8). This offers yet another example of the vulnerability of isolated populations to explain the decline in coral and framework frequency in the southern archipelago^47,56^. Also *Gardineroseris planulata* at Pta. Estrada (Sta. Cruz) disappeared as did several large *Porites lobata* colonies at Punta Pitt (Española)^50,57^.

If the current patterns of ENSO disturbances were to change significantly^7^, it is unclear how dramatic the impact on the long-term connectivity patterns would be. Our modelling data included the ENSO periods 2005/6, 2009/10 and the onset of the 2015/16 event. The overall larval connectivity patterns (Fig. 8) were an integration of both ENSO and non-ENSO effects. Connectivity within the wider region varied and was sensitive to assumptions of pelagic larval duration which, however, is poorly constrained. Detailed mechanisms and patterns of connectivity need more investigation but our model results agree with other studies showing intermittent and variable connectivity of the Galápagos to other Eastern Pacific sites^29^.

### Coral colony sizes and frameworks

Although conditions at both Darwin and Wolf are conducive to reef growth and attainment of large coral sizes, only Wellington Reef at Darwin is a true structural framework reef^38^. Incipient frameworks occur at Wolf within the depth zone of maximum coral size at around 9–17m^25,38^ (Fig. 6). Wellington Reef is ~4m-thick and has undergone accretion for at least 400–600 years^38^ (Fig. 1). Darwin is surrounded by similar coral growth as at Wolf (large, isolated corals on stable boulders, but no continuous reef framework), in areas of comparable morphology (e.g. steep flanks at >40° angle), but only the relatively flat erosional surface between the island and Darwin’s arch allowed for reef framework accretion (Fig. 1). Wellington Reef is situated within the depth zone of maximum coral size (Fig. 6). This does not coincide with the shallowest part of the platform on the island’s windward (SW) side (Fig. 1B,C), with near-constant exposure to strong wave and surge action. Since coral colony sizes generally decrease at <10 m depth and attain maximum sizes in the 9–17 m depth range across all sites (Fig. 6), strong physical control in shallow areas seems evident^38^. Only the flat seafloor on Darwin plateau, sheltered from the most forceful wave action by a shallow ridge (Fig. 1), apparently provides sufficient stable substratum where incipient frameworks can become established and avoid being dislodged by mobile boulders during stormy periods.

### Competitors and predators of corals

Sea urchin abrasion contributes significantly to coral skeleton breakdown, especially if colonies are less calcified and therefore more friable, as is the case in the southern Galápagos^22,31^. Modelling of the dynamics of Wellington Reef confirms that sea urchin bioerosion controls framework growth^58^. Thus, more friable skeletons in the southern islands and higher sea urchin densities there^17,38,58^ (Fig. 7A) indeed contribute to the more rapid and prevalent degradation of reef frameworks and slower recovery in the south^4,25,47^.

### Environmental conditions

Distinct latitudinal gradients in coral reef development in the Galápagos have been described^4,17,22,25,27,28,31,47^ and were substantiated by this study with regards to sea temperature (means, maxima and minima), the range of temperature excursions due to thermocline shoaling, levels of dissolved nutrients, pH, and degree of biotic connectivity. Not one physical factor alone had overriding importance, but analyses suggest interactions of several factors, most importantly minimum sea temperatures, pH and nutrient availability, causing the observed gradients in coral community characteristics. A gradient in water-temperature influences coral growth and therewith framework-development and cover. Thermocline-shoaling^18,19^ seems to be important since it can occur more frequently during the warm season when bleaching is most prevalent (Fig. 9). Not only do such events reduce temperature, they also create nutrient-enriched conditions (Fig. 10) that can assist coral recovery after bleaching^59,60^. Thermocline shoaling prevented coral mass mortality during the 2015/16 ENSO event^61^. A north-south gradient in pH and carbonate saturation state^21,22^ leads to lower rates of coral calcification and precipitation of carbonate cements in the south. Coral skeletons at the southern islands harbored more eroding polychaete worms and bivalve mollusks, and experienced more sea urchin grazing than in the north (Fig. 7), resulting in more bioerosion. Also, a combination of lowered pH and high PO_4_^3−^, typical of waters in the south, can increase mortality of corals under stress^62^. High incidences of coral diseases were observed at Darwin, Wolf and Marchena between 2005–7^63^ (see also Fig. 5), but tissue loss subsequently subsided and increased again only during the period of invasion by *Caulerpa* spp. This coral resilience in the northern islands may be due in large part to a more benign thermal and chemical environment. Corals in the southern Galápagos face greater environmental challenges than those in the north. For example, cooler water, lower pH, more competitors and bioeroders. They are more prone to simultaneous region-wide disturbance due to larval connectivity primarily to populations in similar disturbance regime and environmental setting. This situation disadvantages them sufficiently to deny them filling all available habitat^64^ and results in lower diversity, space cover and generally smaller colonies.

In conclusion, Galápagos corals occur in a highly variable physical and chemical environment that poses significant challenges to survival. Strongly influenced by ENSO events, region-wide mortality has repeatedly been observed (1982/3, 1997/8). The northern islands (Darwin and Wolf) support richer scleractinian coral communities that cover more space and consist of generally larger colonies, and have exhibited more rapid recovery after disturbance events. This is due to generally warmer and less acidic waters, thermocline shoaling during the warm season and during ENSO activity that helps prevent bleaching, and importantly, strong downstream larval connectivity with southern sites. Southern sites have less coral cover and generally smaller coral colonies (except *Pavona* spp.) with fewer recruits. Larval connectivity is among southern islands and directed primarily towards the northern islands, but not in the opposite direction. Not one single environmental factor was of overriding importance for coral community differentiation, but an interaction of sea temperature, pH (declining N-S) and nutrient regime (increasing N-S). The northern islands of Darwin and Wolf are clearly situated in a privileged position for coral survival and are expected to maintain the best coral growth with the highest recovery potential following disturbances. The two northern islands of Darwin and Wolf are therefore of prime conservation importance for corals in the Galápagos Archipelago.

## Supplementary information

Supplementary Information
