# Supplementary material for: Some environmental and biological determinants of coral richness, resilience and reef building in Galápagos (Ecuador)
Source: Sci Rep. 2019 Jul 16;9:10322. doi: 10.1038/s41598-019-46607-9 (PMC6635370; doi:10.1038/s41598-019-46607-9)
Supplement: Supplementary file 1 — Supplementary Information [file 41598_2019_46607_MOESM1_ESM.pdf]

Supplementary Information

Some environmental and biological determinants of coral richness, resilience, and reef building in Galápagos (Ecuador)

Bernhard Riegl[1,corresponding author], Matthew Johnston[1], Peter W. Glynn[2], Inti Keith[3], Fernando Rivera[4], Mariana Vera-Zambrano[5], Stuart Banks[5], Joshua Feingold[1], Peter J. Glynn[6]

1) Halmos College of Natural Science and Oceanography, Nova Southeastern University, 8000 N Ocean Drive, Dania Beach, Florida 33004, USA. Correspondence to: rieglb@nova.edu
2) Rosenstiel School of Marine and Atmospheric Science, University of Miami, 4600 Rickenbacker Causeway, Miami, Florida 33149-1098, USA.
3) Fundación Charles Darwin, Charles Darwin Research Station, Puerto Ayora, Santa Cruz Island, 200350 Galápagos, Ecuador.
4) Instituto Nazca de Investigaciones Marinas, Av 8va 1615 Calle 20, Salinas, 241550 Santa Elena, Ecuador.
5) Conservation International, Puerto Ayora, Santa Cruz Island, 200350 Galápagos, Ecuador.
6) Crane Country Day School, 1795 San Leandro Lane, Santa Barbara, CA 93108, USA.

Abstract: Throughout the Galápagos, differences in coral reef development and coral population dynamics were evaluated by monitoring populations from 2000-2019, and environmental parameters (sea temperatures, pH, NO_3^-, PO_4^{3-}) from 2015-19. The chief goal was to explain apparent coral community differences between the northern (Darwin and Wolf) and southern (Sta. Cruz, Fernandina, San Cristóbal, Española, Isabela) islands. Site coral species richness was highest at Darwin and Wolf. In the three most common coral taxa, a declining North (N)-South (S) trend in colony sizes existed for *Porites lobata* and *Pocillopora* spp., but not for *Pavona* spp.. Frequent coral recruitment was observed in all areas. Algal competition was highest at Darwin, but competition by bioeroding sea urchins and burrowing fauna (polychaete worms, bivalve mollusks) increased from N to S with declining coral skeletal density. A biophysical model suggested strong connectivity among southern islands with weaker connectivity to Wolf and even less to Darwin. Also, strong connectivity was observed between Darwin and Wolf, but from there only intermittently to the south. From prevailing ocean current trajectories, coral larvae from Darwin and Wolf drift primarily towards Malpelo and Cocos Islands, some reaching Costa Rica and Colombia. Mean temperature, pH, and PO_4^{3-} declined from N to S. Strong thermocline shoaling, especially in the warm season, was observed at most sites. A single environmental factor could not explain the variability in observed coral community characteristics, with minimum temperature, pH and nutrients the strongest determinants. Thus, complex environmental determinants combined with larval connectivity patterns may explain why the northern Galápagos Islands (Darwin, Wolf) have higher coral richness and cover and also recover more rapidly than central/southern islands after region-wide disturbances. These northern islands are therefore potentially of critical conservation importance as important reservoirs of regional coral biodiversity and source of larvae.

This Supplementary Information provides tables, graphs and data to provide additional information to that presented in the main body of our paper. The information provided here may be helpful for further interpretation or understanding of the necessarily brief Results section. Data and graphics shown here strengthen the points made in the paper, but were excluded to avoid clutter from too many presentations.

a) Sample site details

Sampling periods and data collected between 2000 and 2019 provided the basis for the present paper and an overview is presented below.

Sampling sites were the following:

Wolf: Bahia Tiburón and Anchorage Bay.

Darwin: Wellington Reef, Anchorage Bay, Hidden Reef.

Marchena: Punta Espejo.

Santiago: Sullivan Bay.

*Bartolom*é: north-shore, ~ 1km E of Tower Rock.

Baltra/Sta. Cruz: Western entry to Itabaca Channel, Eastern part of Itabaca channel.

Santa Fé: NE Anchorage = Barrington Bay (inside Islote Black).

San Cristóbal: Punta Pitt, Manzanillo.

Española: Gardner Island, Osborn (Gayle) and Xarifa (Tortuga) Island, Gardner Bay.

Floreana – coral sites: Corona del Diablo, Tres Cuevitas, Water chemistry samples only: Luz del Dia and La Botella.

Isabela: coral sites: Concha de Perla lagoon, Caseta, Urvina Bay; Water chemistry samples only: Punta Moreno, Playa Negra, Los Cañones.

Fernandina: Punta Espinosa (few individual corals), Cabo Douglas (water chemistry and temperature only, no corals encountered).

Table SI-1: *Overview of more recent sampling across the Galapagos archipelago and types of data upon which the present paper is based:*

			Phototransects	Line Transects	Water Chemistry	in situ size measurements	bathymetry	Temperature loggers
March	2000	Wolf, Darwin, Floreana, San Cristobal		■		■		
March	2007	Wolf, Darwin, Seymour, Marchena		■		■		
March	2012	Wolf, Darwin, Marchena, Floreana, Espanola,Isabela,	■	■		■		
April	2013	Baltra,Sta.Cruz, Sta. Fe, Espanola, Floreana, San Cristobal, Isabela		■		■		
July	2014	Floreana						■
March	2015	Wolf, Darwin	■			■		■
March	2016	Wolf, Darwin	■		■			■
November	2016	Isabela, Fernandina, Wolf, Darwin	■		■	■	■	■
March	2017	Wolf, Darwin	■		■	■	■	■
March	2018	Wolf, Darwin	■		■	■		■
March	2019	Wolf, Darwin, Marchena, Floreana, Espanola,Isabela, Fernandina, Santiago,Bartolome	■		■	■	■	■

b) Coral occurrence across the archipelago

This section of the Results discusses differences in species richness among the islands and also differences in occurrence. An nMDS is presented, based on the differential occurrence of species. Table SI-2 shows coral occurrence as documented in samples across the Galápagos Archipelago, pooled for each island. Note that this may not be an exhaustive list of all hermatypic corals occurring at each location and that concerted searches are likely to yield more species at some sites than listed here.

Table SI-2: *Galápagos zooxanthellate coral species and their records in the samples for this study (2000-2018).* Porites evermanni *is a cryptic species that cannot be identified reliably by morphology alone. It has been recorded from Darwin and Espanola*[1]*, therefore, we suspect that it also makes up a part of our sample identified as* Porites lobata*. +=no longer recorded, *=now very rare. The numerically dominant species at the sampling sites is indicated in bold letters. At Floreana, sites are dominated either by unattached corals (near Corona del Diablo*, P. stellata, D. distorta*) or attached, framework-forming corals (near Tres Cuevitas* P. clavus*).*

	**Darwin**	**Wolf**	**Marchena**	**Santiago**	**Bartolomé**	**Baltra/S.Cruz**	**Santa Fe**	**San Cristóbal**	**Española**	**Floreana**	**Isabela - E**	**Isabela - W**	**Fernandina**
Psammocora stellata	X	X	**X**	X	X	X	X	**X**	X	**X**	**X**		
Pocillopora verrucosa	X	X	X	X	X	X		X	X	X			
Pocillopora damicornis	X	X	X	X							**X**		
Pocillopora meandrina	X	X		X					X	X			
Pocillopora capitata	X	X											
Pocillopora eydouxi	X	X											
Pocillopora effusus	X	X			X					X			
Pocillopora inflata	X	X		X					X	X			
Pocillopora ligulata	X	X											
Pocillopora woodjonesi	X	X											
Pavona varians	X	X		X	X	X	X	X		X			
Pavona chiriquiensis	X	X		X	X	X	X	X	X	X			
Pavona clavus	X	**X**		**X**	**X**	**X**		**X**	X	**X**	X		
Pavona gigantea	X	X		X	X	X		X	X	X		X	**X**
Pavona maldivensis	X	X											
Gardineroseris planulata	X	X				+				X			
Leptoseris scabra	X	X											
Leptoseris sp. 1	X	X											
Cycloseris curvata	+								+	X*			
Diaseris distorta										**X**			
Porites lobata	**X**	**X**	X	X	X	X		+	X	X	X	**X**	
Porites evermanni	X								X				
TOTAL	20	19	4	10	8	7	3	6	9	13	4	2	1

Also *Psammocora profundacella* was recorded in the southern islands[2], but not encountered in the present study.

Table SI-3: *ANOVA table comparing means of coral richness observed across all sites across all survey years.*

```
Call:
aov(formula = spec$Species ~ as.factor(spec$Code))
Residuals:
    Min      1Q  Median      3Q     Max
-2.3333 -0.6667  0.0000  0.3333  2.2000
Coefficients:
```

	Estimate	Std. Error	t value	Pr(>|t|)	
(Intercept)	17.3333	0.5048	34.340	< 2e-16	***
as.factor(spec$Code)2	-0.3333	0.7138	-0.467	0.645	
as.factor(spec$Code)3	-13.3333	1.0095	-13.208	8.95e-13	***
as.factor(spec$Code)4	-14.5833	0.7981	-18.273	5.63e-16	***
as.factor(spec$Code)5	-16.6667	0.8743	-19.064	< 2e-16	***
as.factor(spec$Code)6	-8.3333	1.3355	-6.240	1.58e-06	***
as.factor(spec$Code)7	-10.3333	1.3355	-7.738	4.29e-08	***
as.factor(spec$Code)8	-10.3333	0.8743	-11.819	9.93e-12	***
as.factor(spec$Code)9	-15.3333	1.0095	-15.189	3.95e-14	***
as.factor(spec$Code)10	-12.3333	1.3355	-9.235	1.56e-09	***
as.factor(spec$Code)11	-6.6667	0.8743	-7.625	5.57e-08	***
as.factor(spec$Code)12	-6.5333	0.7487	-8.727	4.65e-09	***

```
Signif. codes:  0 ‘***’ 0.001 ‘**’ 0.01 ‘*’ 0.05 ‘.’ 0.1 ‘ ’ 1
Residual standard error: 1.236 on 25 degrees of freedom
Multiple R-squared:  0.9725, Adjusted R-squared:  0.9604
F-statistic: 80.37 on 11 and 25 DF,  p-value: < 2.2e-16
```

c) Trends in coral colony sizes across the archipelago

Figure 3 in the main text provides a contour plot that conveniently shows size-trends in hermatypic coral colonies across the archipelago. These contour plots use the median coral sizes per island as shown in Figure SI-1 as underlying data.

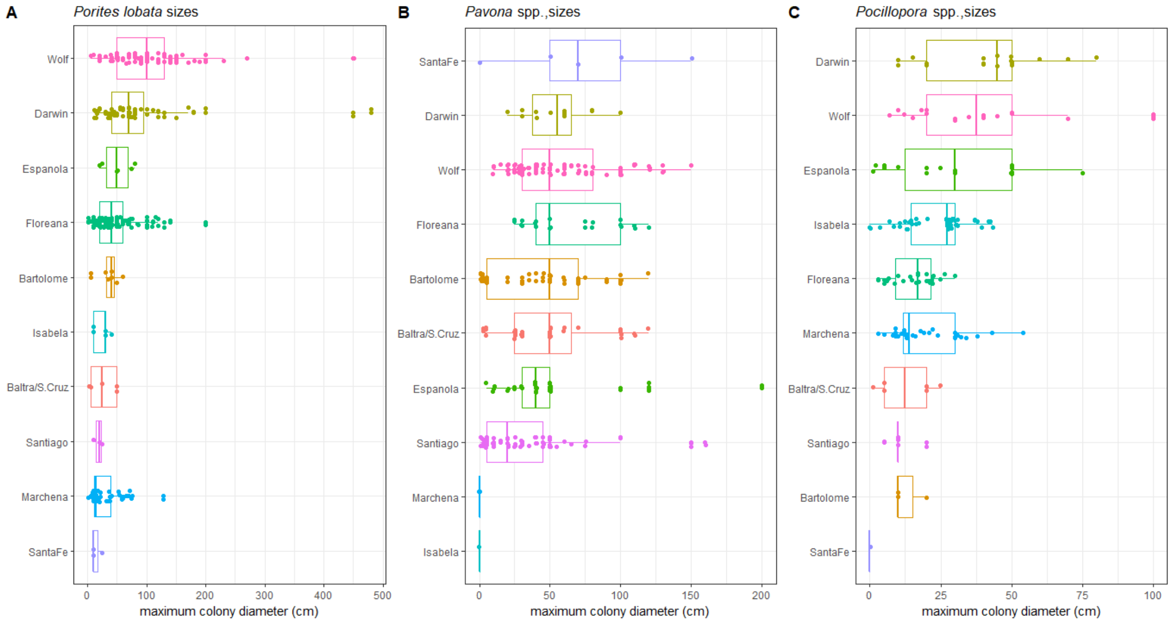

Figure SI-1: *Boxplots of maximum horizontal coral diameter, arranged from greatest to smallest median sizes, across all Galápagos sites. The jitterplots show the position of actual datapoints. Since sampling areas and sampling effort were comparable among islands, greater point-density in any site indicates a greater relative frequency of the coral. Each jittered point indicates one sample. The y-axis label "maximum colony diameter" refers to the fact that measurements of the longest horizontal axis of colonies was used to evaluate size distribution.*

Table SI-4: *ANOVA table for test of* P. lobata *sizes among the islands.*

```
Call:
aov(formula = log(dat1$Diam1) ~ as.factor(dat1$Site))
Residuals:
    Min      1Q  Median      3Q     Max
-3.5206 -0.5089  0.1204  0.5364  2.0386
Coefficients:
```

	Estimate	Std. Error	t value	Pr(>|t|)	
(Intercept)	4.1352	0.1047	39.512	< 2e-16	***
as.factor(dat1$Site)Wolf	0.2442	0.1403	1.740	0.082700	.
as.factor(dat1$Site)Marchena	-1.1414	0.1556	-7.333	1.67e-12	***
as.factor(dat1$Site)Santiago	-1.2961	0.4909	-2.640	0.008667	**
as.factor(dat1$Site)Bartolome	-0.7137	0.3310	-2.157	0.031746	*
as.factor(dat1$Site)Baltra/S.Cruz	-1.3850	0.3860	-3.588	0.000382	***
as.factor(dat1$Site)SantaFe	-1.5271	0.4909	-3.111	0.002023	**
as.factor(dat1$Site)Espanola	-0.3455	0.3549	-0.973	0.331051	
as.factor(dat1$Site)Floreana	-0.6146	0.1283	-4.788	2.52e-06	***
as.factor(dat1$Site)Isabela	-1.1159	0.3860	-2.891	0.004086	**

```
Signif. codes:  0 ‘***’ 0.001 ‘**’ 0.01 ‘*’ 0.05 ‘.’ 0.1 ‘ ’ 1
Residual standard error: 0.8307 on 338 degrees of freedom
Multiple R-squared:  0.2816, Adjusted R-squared:  0.2625
F-statistic: 14.72 on 9 and 338 DF,  p-value: < 2.2e-16
```

Table SI-5: *ANOVA table for test of* Pocillopora *spp. sizes among the islands.*

```
Call:
aov(formula = log(dat3$Diam1) ~ as.factor(dat3$Site))
Residuals:
    Min      1Q  Median      3Q     Max
-2.9393 -0.4374  0.1981  0.4620  1.3782
Coefficients:
```

	Estimate	Std. Error	t value	Pr(>|t|)	
(Intercept)	3.55045	0.17983	19.744	< 2e-16	***
as.factor(dat3$Site)Wolf	-0.03005	0.23943	-0.126	0.900277	
as.factor(dat3$Site)Marchena	-0.77653	0.22252	-3.490	0.000622	***
as.factor(dat3$Site)Santiago	-1.24786	0.37721	-3.308	0.001156	**
as.factor(dat3$Site)Bartolome	-1.01681	0.46431	-2.190	0.029948	*
as.factor(dat3$Site)Baltra/S.Cruz	-1.47891	0.35208	-4.201	4.37e-05	***
as.factor(dat3$Site)Espanola	-0.61115	0.25076	-2.437	0.015875	*
as.factor(dat3$Site)Floreana	-0.97354	0.23126	-4.210	4.21e-05	***
as.factor(dat3$Site)Isabela	-0.44716	0.21242	-2.105	0.036816	*

```
Signif. codes:  0 ‘***’ 0.001 ‘**’ 0.01 ‘*’ 0.05 ‘.’ 0.1 ‘ ’ 1
Residual standard error: 0.7414 on 163 degrees of freedom
Multiple R-squared:  0.2253, Adjusted R-squared:  0.1873
F-statistic: 5.926 on 8 and 163 DF,  p-value: 1.16e-06
```

Table SI-6: *ANOVA table for test of* Pavona *spp. sizes among the islands*

```
Call:
aov(formula = log(dat2$Diam1) ~ as.factor(dat2$Site))
Residuals:
    Min      1Q  Median      3Q     Max
-3.1472 -0.5572  0.2049  0.7158  2.2068
Coefficients:
```

	Estimate	Std. Error	t value	Pr(>|t|)	
(Intercept)	3.895014	0.297707	13.083	< 2e-16	***
as.factor(dat2$Site)Wolf	-0.005609	0.317357	-0.018	0.98591	
as.factor(dat2$Site)Santiago	-1.026595	0.321246	-3.196	0.00154	**
as.factor(dat2$Site)Bartolome	-0.747810	0.327058	-2.286	0.02289	*
as.factor(dat2$Site)Baltra/S.Cruz	-0.329206	0.344988	-0.954	0.34068	
as.factor(dat2$Site)SantaFe	0.549067	0.595415	0.922	0.35715	
as.factor(dat2$Site)Espanola	-0.240003	0.346281	-0.693	0.48876	
as.factor(dat2$Site)Floreana	0.145322	0.373196	0.389	0.69724	

```
Signif. codes:  0 ‘***’ 0.001 ‘**’ 0.01 ‘*’ 0.05 ‘.’ 0.1 ‘ ’ 1
Residual standard error: 1.031 on 317 degrees of freedom
Multiple R-squared:  0.1556, Adjusted R-squared:  0.137
F-statistic: 8.348 on 7 and 317 DF,  p-value: 2.304e-09
```

An Analysis of Covariance (ANCOVA) assuming depth and site (=island) as independent factors, with coral diameter as variate, suggested that site had a significant influence while depth failed significance (Table SI-7). The model assuming site:depth interaction was significant (Table SI-8).

Table SI-7: *Terms in the ANCOVA model of coral diameter as a function of depth and island.*

```
Model:
log(dat$Diam1 + 1) ~ depth + dat$Site
```

	Df	Sum of Sq	RSS	AIC	F value	Pr(>F)	
Full model			721.35	-150.549			
Without depth	1	2.971	724.32	-148.940	3.5713	0.05912	.
Without Site	9	141.884	863.23	-10.894	18.9481	< 2e-16	***

```
---
Signif. codes:  0 ‘***’ 0.001 ‘**’ 0.01 ‘*’ 0.05 ‘.’ 0.1 ‘ ’ 1
```

Table SI-8: *Comparison of the full ANCOVA model with one where the interaction is dropped and F-test comparing the residual sum of squares of both. The interaction is significant.*

```
Model:
log(dat$Diam1) ~ depth * dat$Site
```

	Df	Sum of Sq	RSS	AIC	F value	Pr(>F)	
Full model			749.76	-90.553			
Without interaction	9	27.394	777.15	-77.297	3.4548	0.000338	***

```
---
Signif. codes:  0 ‘***’ 0.001 ‘**’ 0.01 ‘*’ 0.05 ‘.’ 0.1 ‘ ’ 1
```

d) Larval connectivity within the Galápagos

Larval trajectories were evaluated during ENSO and non-ENSO events. Patterns of larval dispersal in the Galápagos archipelago during ENSO years (2005, 2010) were similar with regards to south-north dispersal. However, during ENSO years, more westward trajectories were observed and fewer larvae reached Central and South America (Fig. SI-2).

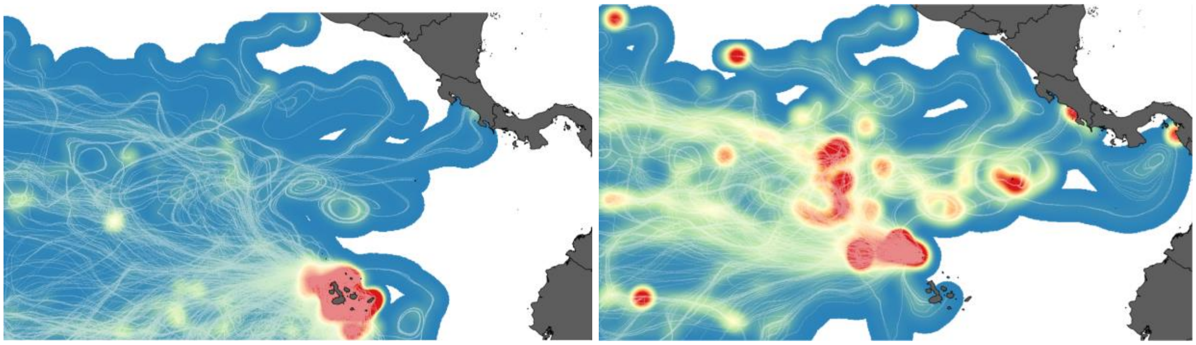

Fig. SI-2: *Larval connectivity patterns within the Galápagos Archipelago and adjacent Tropical Eastern Pacific in the ENSO years 2005 and 2010 (combined). Heat colors show number of larvae encountered by area (red=most, blue=least), yellow lines trace selected individual larval trajectories.*

e) Temperature as drivers of coral dynamics

This section of the paper shows results of analyses that relate coral sizes to means, maxima and minima in the HadISST[3] datasets. We obtained the HadISST 1x1 geographic degree temperature tiles for the Galápagos, which provide a synthetic temperature record from 1870 to the present (from https://coastwatch.pfeg.noaa.gov). To evaluate how reliably this synthetic dataset reflected on-the-ground conditions, we compared it to a 50-plus year dataset of *in situ* temperature recordings that was available from Puerto Ayora[4] (downloaded in 2013, dataset no longer publicly available) We examined the correlation of mean monthly SST in the time-window 1981-2012 of the synthetic HadISST datasets against that of the Puerto Ayora data. HadISST data correlated highly with the *in situ* data over the comparison interval (R^2=0.88). HadISST data closely tracked locally observed temperature dynamics, however, *in situ* data were shifted downward by ~ 1°C, a result of the *in situ* thermo-sensor situated in deeper water than the ocean’s surface skin (as considered by SST temperature models) and cool ground water percolating into the area. Extremes in HadISST were of smaller magnitude than *in situ* records (Fig. SI-3).

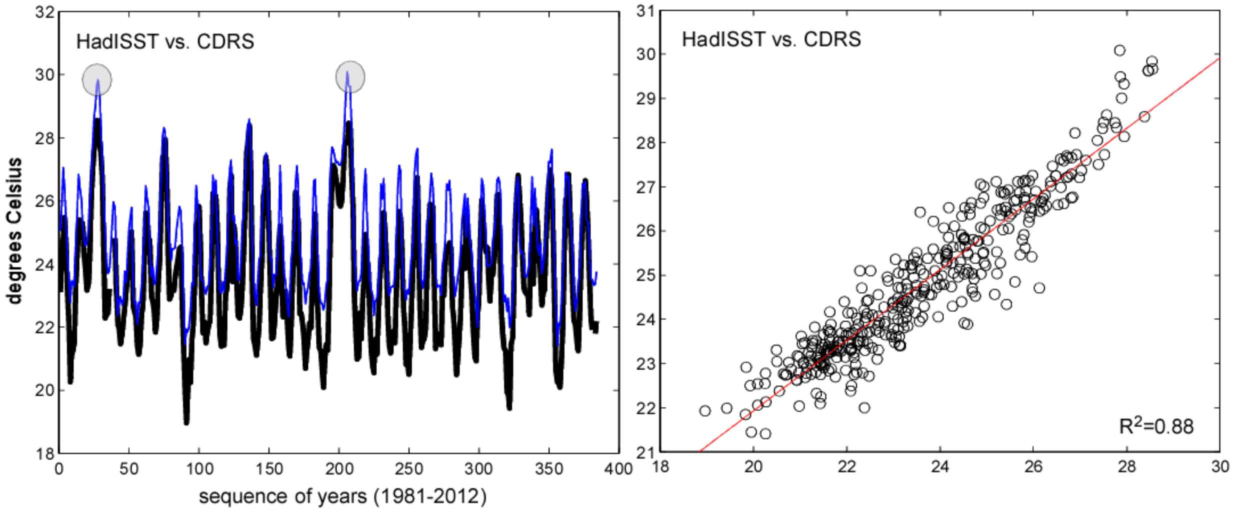

Figure SI-3: *HadISST temperature dataset agrees with in-situ measurements at Puerto Ayora (CDRS=Charles Darwin Research Station), Sta. Cruz, Galápagos (5). Grey circles denote the 1983 and 1998 ENSO (El Niño Southern Oscillation) events that led to significant coral mortality. Black line=in situ data, blue line=HadISST data. Panel to the right shows correlation.*

Figure SI-4 shows the HadISST data for two extremes, Darwin in the tropical north and Isabela in the area of strongest upwelling, as reference.

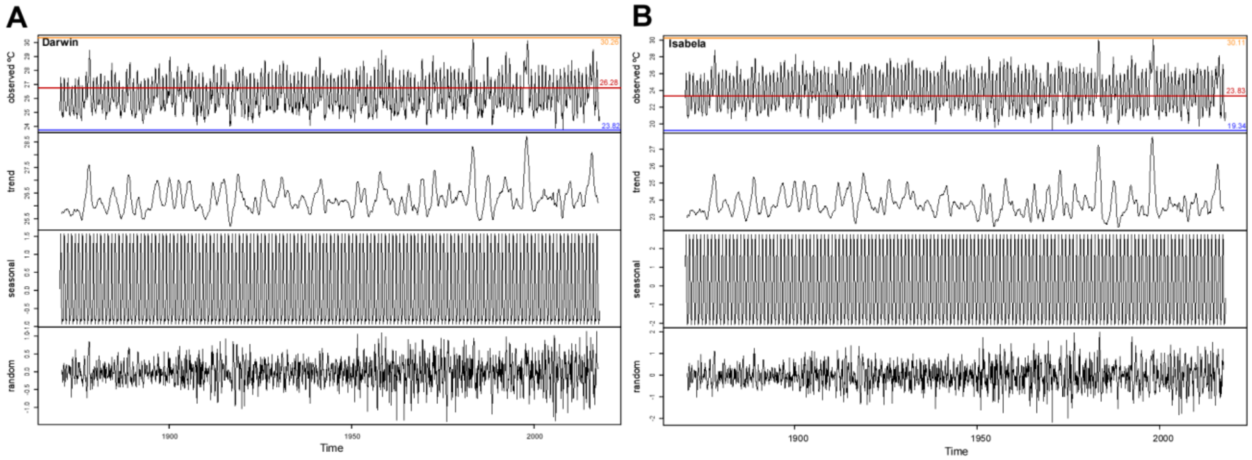

Figure SI-4*: Classical decomposition of an additive timeseries. Long-term temperature differences between the warmest and coldest regions in the Galápagos based on monthly mean values of HadISST 1x1 geographic degree tiles. (A) Mean SST at Darwin and Wolf is 3ºC higher than at Isabela (both areas dominated by* Porites lobata*, but zooxanthellate corals uncommon at Isabela). Time-series decompositions show the original data, the trend as a 12-point moving average, the seasonal and the random components. The upper panels show the ENSO disturbances of 1982/3, 1997/8 and 2015/16 particularly clearly as upward spikes in trend and observed temperatures. The red line in the uppermost panels is mean SST, orange is maximum, blue is minimum.*

Fig. SI-5 A shows temperature records with 30-minute frequency along a gradient from the northernmost to the westernmost to the southernmost islands in the Galápagos. While Fernandina (wetsrenmost) had the lowest mean temperature, Floreana (southernmost) showed more thermocline shoaling activity. While Wolf (northernmost) showed strong variability, the amplitude of daily temperature swings was much lower. Daily mean temperatures (24-hour mean, Fig. SI-5B) masked many details of rapid temperature oscillations, but the record of daily means at Darwin still showed the importance of frequent thermocline shoaling. A record of daily means at Academy Bay on St. Cruz, showed equally frequent oscillations, however with much smaller amplitude than at Wolf. This may have been due to the thermo-monitoring site being situated inside a shallow bay. At Darwin, Wellington Reef (main text Fig. 1 B,C) borders several submarine ravines that can funnel below-thermocline waters, delivered by internal tidal bores or internal waves, directly to the reef. Thermocline shoaling events are not synchronous across the archipelago and differences in timing can be detected both in the 30-minute as well as the daily-means datasets.

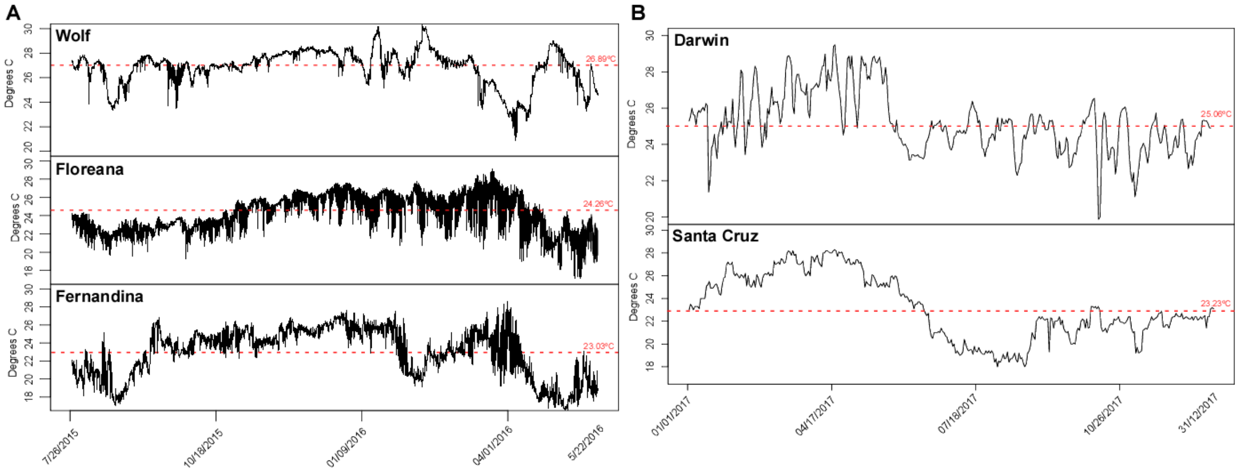

Figure SI-5: *A variety of patterns are observed in the thermal environment of Galápagos corals: (A) hourly temperatures at Wolf (10m depth), Floreana (10m depth) and Cabo Douglas (Fernandina, 10m depth). No zooxanthellate corals were observed at Cabo Douglas, which is one of the coldest sites with active upwelling, but Glynn and Wellington (1983) observed* Pocillopora *frameworks at Punta Espinosa on Fernandina in 1979. (B) Daily means show that although Darwin (12m depth) is warmer than Santa Cruz (5m depth), thermocline shoaling is more frequent and more pronounced at Darwin. Sheltered Academy Bay at Sta. Cruz presently has no framework reef, but incipient frameworks were described at this site by Glynn and Wellington (1983).*

f) Multivariate coral community determination by environmental variables

Strong univariate relationships existed in the interplay of environmental and biological factors with species richness and framebuilding in the groupings of islands (as suggested by main text Fig. 2). The significant differences among the island groups in several variables are illustrated below (Fig. SI-6).

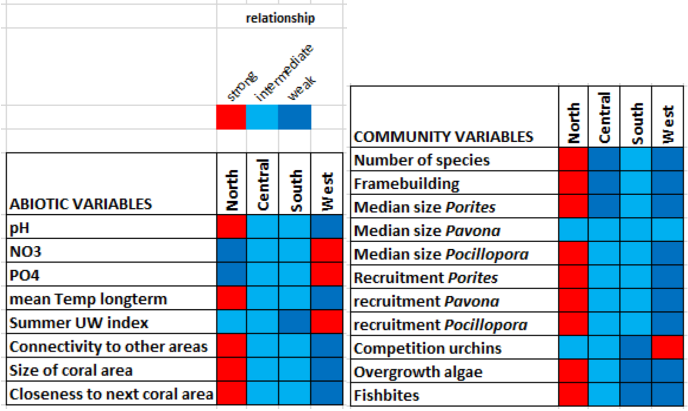

Figure SI-6: *Overview of univariate statistical relationships between abiotic and community variables and the island groups as defined in Fig. 2. Significance levels and types of analyses can be found in the preceding text. A contrast red (highest values) -dark blue (lowest values) signifies highly significant differences (if resultant of ANOVA) or a significant correlation (regression analyses). Contrasts to light blue (medium values) do not necessarily imply significance to either red or dark blue.*

Citations in SI:

1. Boulay, J.N., Hellberg, M.E., Cortes, J., Baums, I.B. Unrecognized coral species diversity masks differences in functional ecology. *Proceedings of the Royal Society B* doi:10.1098/rspb.2013.1580, (2013).
2. Glynn, P.W., Colley, S.B., Maté, J.L., Baums, I.B. Feingold, J.S., Cortés, J., Guzman, H.M., Afflerbach, J.C., Brandtneris, V.W., Ault, J.S. Reef coral reproduction in the equatorial Eastern Pacific: Costa Rica, Panama, and the Galapágos Islands (Ecuador). VII. Siderastaeidae, *Psammocora stellata*, *Psammocora profundacella*. *Marine Biology* doi: 10.1007/s00227-012-1979-5, (2012).
3. Rayner, N.A., Parker, D.E., Horton, E.B., Folland, C.K., Alexander, L.V., Rowell, D.P., Kent, E.C., Kaplan, A. Global analysis of sea surface temperature, sea ice, and night marine air temperature since the late nineteenth century. *Journal of Geophysical Research* **108** D14, 4407, doi: 10.1029/2002JD002670, (2003).
4. Charles Darwin Research Station. http://www.darwinfoundation.org/datazone/ (2013) Date of access: 15/03/2013.
